# Investigating the Feasibility of Preparing Metal–Ceramic Multi-Layered Composites Using Only the Aerosol-Deposition Technique

**DOI:** 10.3390/ma14164548

**Published:** 2021-08-13

**Authors:** Matej Sadl, Urban Tomc, Hana Ursic

**Affiliations:** 1 Electronic Ceramics Department, Jožef Stefan Institute, Jamova Cesta 39, 1000 Ljubljana, Slovenia; matej.sadl@ijs.si; 2 Jožef Stefan International Postgraduate School, Jamova Cesta 39, 1000 Ljubljana, Slovenia; 3 Laboratory for Refrigeration and District Energy, Faculty of Mechanical Engineering, University of Ljubljana, Aškerčeva Cesta 6, 1000 Ljubljana, Slovenia; urban.tomc@fs.uni-lj.si

**Keywords:** aerosol deposition, multilayers, interdigitated aluminium electrodes, alumina insulating layers

## Abstract

The preparation of metal–ceramic layered composites remains a challenge due to the incompatibilities of the materials at the high temperatures of the co-firing process. For densification, the ceramic thick-film materials must be subjected to high-temperature annealing (usually above 900 °C), which can increase the production costs and limit the use of substrate or co-sintering materials with a low oxidation resistance and a low melting point, such as metals. To overcome these problems, the feasibility of preparing dense, defect-free, metal–ceramic multilayers with a room-temperature-based method should be investigated. In this study, we have shown that the preparation of ceramic–metal Al_2_O_3_/Al/Al_2_O_3_/Gd multilayers using aerosol deposition (AD) is feasible and represents a simple, reliable and cost-effective approach to substrate functionalisation and protection. Scanning electron microscopy of the multilayers showed that all the layers have a dense, defect-free microstructure and good intra-layer connectivity. The top Al_2_O_3_ dielectric layer provides excellent electrical resistance (i.e., 7.7 × 10^12^ Ω∙m), which is required for reliable electric field applications.

## 1. Introduction

Thick-film multilayer technology is of great value in the electronics industry. It enables the development and design of a wide variety of products, such as microsystems, electronic circuit boards and micro-electromechanical systems. The fabrication of conventional thick-film multilayers involves well-developed technologies, i.e., tape-casting and screen-printing, followed by a co-firing process at elevated temperatures [1]. The co-firing process poses many problems in the fabrication of metal–ceramic multilayers. First, the mismatch of firing temperatures significantly limits the choice of compatible materials, as metals require much lower temperatures than ceramics. Second, a high-temperature process facilitates metal oxidation and diffusion between the layers, which can lead to functional degradation, posing major challenges in material selection [2]. On the other hand, a high-temperature firing process can be avoided by using aerosol deposition (AD), which is a room-temperature spray-coating method for producing dense, micrometre-thick films. It requires dry powders of approximately micrometre-sized particles that are mixed with a carrier gas to form an aerosol [3]. In the deposition process, the aerosol jet hits the substrate with a high kinetic energy under vacuum conditions. The AD method is often referred to as a simple and rapid deposition method capable of producing very dense films without adding any external thermal energy to the aerosol or deposited films [4]. The high density of the deposited materials is achieved by the hammering effect of powder particles colliding with the surface of the substrate, fragmenting and re-bonding [4,5,6]. This deposition mechanism is referred to as a room-temperature, impact-consolidation mechanism [6]. AD is a unique approach to the deposition of ceramic coatings at room temperature, which means the vast majority of research has been conducted on ceramic coatings, such as simple oxides (Al_2_O_3_ [7], TiO_2_ [8], Y_2_O_3_ [9]), perovskites (Pb(Zr,Ti)O_3_ [10], BaTiO_3_ [11]) and others (AlN [12], hydroxyapatite [13]). However, AD is not limited to ceramics; metals, glasses or polymers can also be deposited. The deposition of metal layers is often associated with other spray-coating techniques, such as cold spray, which requires heating of the carrier gas (up to 700 °C). In contrast to cold spray, the AD process is much more advantageous for the deposition of metal layers, since the deposition at room temperature avoids deleterious oxidation, decomposition and thermal shock to the coating and the substrate [14]. However, the AD of metal films is still poorly understood, since in the AD community research is mainly focused on ceramic deposition. The deposition of metals poses a great challenge because the modification of powder parameters (e.g., particle size distribution, morphology and agglomeration state) is much more challenging for metals compared to ceramic materials. For example, ceramic powders can be coarsened very easily by partial sintering, which only requires heating the powder at ambient atmosphere. In addition, the brittleness of ceramic powders allows straightforward particle size reduction and de-agglomeration in a ball milling process. On the other hand, metal powders are much more susceptible to oxidation during heat treatment and their ductile behaviour significantly complicates the milling process. The first dense metal films prepared using AD at room temperature were reported less than 10 years ago [15]. The development of metal films by AD is still in its early stages. Thus far, AD has been used to deposit metal films such as Cu [16,17], Ag [15,18], Fe [19,20] and Fe-based amorphous alloys [21]. In this study, we have deposited metal layers of Al, which has not yet been reported.

The AD method is mostly used for the fabrication of single layers [4]. Although AD is considered as an additive technology, only a few multilayers have been demonstrated thus far. There are few reports involving the fabrication of multilayers using a combination of different deposition methods. For example, functional ceramic layers are deposited by AD and conductive metal electrode layers are deposited by physical vapour deposition [22,23]. Such an approach can pose difficulties because the metal layers deposited via physical vapour deposition are very thin and their properties degrade after an additional AD of ceramic layers on top. The impact of the ceramic powder on the metal layer is particularly problematic, leading to roughening of the metal–ceramic interface and a potential connectivity loss of the metal layer. There are few reports dealing with the fabrication of multilayers using the AD method. Simple structures consist of ceramic–ceramic [24] or metal–metal multilayers [20]. Thus far, only Leupold et al. [19] have fabricated a ceramic–metal multilayer structure. In this report, multiple thick-film layers of Al_2_O_3_ and Fe were interchangeably deposited, but the microstructural analysis again revealed the connectivity loss of the metal layers. It is obvious that using the AD method to build a metal–ceramic multilayer without structural defects is still a challenge.

In this investigation, we look at the possibility of fabricating a laminated metal–ceramic composite, i.e., a multilayer with a good intra-layer connectivity, using only the AD method. Ceramic alumina (Al_2_O_3_) and metallic aluminium (Al) powders were selected for the deposition due to their low price, high abundance and because they are one of the most commonly used dielectric and electrically conductive materials, respectively. Al_2_O_3_ is often used as an insulating and protective coating, while Al is used as an electrode material. In our previous report we showed that an Al_2_O_3_ thick film deposited on a Gd substrate provides an excellent electrical insulating layer [25]. Based on this, we used AD to build a metal–ceramic multilayer structure with an inter-digitated electrode layer to add functionality to the system. The deposited Al_2_O_3_/Al/Al_2_O_3_ multilayers on the Gd substrate provide an excellent starting point for the development of future electrowetting-on-dielectric devices, such as those used as thermal switches in the magnetocaloric cooling process [26,27] or potentially in any other solid-state, fluidic or mechanical thermal control devices [28,29]. In this study, we have shown that multilayer fabrication using AD is feasible and represents a simple, reliable and cost-effective approach to add functionality and protection to existing substrates.

## 2. Materials and Methods

In the AD, a raw Al powder (99.96 %, 2 HPC, Toyal Europe, Accous, France) and an Al_2_O_3_ powder (99.8 %, A 16 SG, Almatis, Ludwigshafen, Germany) were used. The Al_2_O_3_ powder was thermally treated in a chamber furnace (Custom-made, Terna, Ljubljana, Slovenia) at 1150 °C for 1 h (with 5 K∙min^−1^ heating and cooling rates) and additionally milled in a planetary ball mill (PM400, Retsch, Haan, Germany) at 200 min^−1^ for 5 h using Al_2_O_3_ milling jar and yttria-stabilised-zirconia milling balls with diameters of 3 mm in iso-propanol as a liquid medium. The thermal treatment and the subsequent ball milling of the Al_2_O_3_ powder are necessary to achieve suitable particle size distribution for efficient AD [25]. In the case of Al, the as-received powder already resulted in successful film deposition. Therefore, no further powder modification was required. Prior to the AD, both powders were sieved through an 80-micrometre mesh and vacuum dried for 12 h at 100 °C and at 10 mbar. The AD apparatus was provided by InVerTec e.V., Bayreuth, Germany. Commercial gadolinium foils (Metall Rare Earth Limited, Hong Kong) were used as the substrate material. The process parameters used during the AD are shown in Table 1. The number of scans was adjusted to achieve the desired film thickness.

Particle size analyses of the raw Al powder and treated Al_2_O_3_ powders were performed using a light-scattering laser granulometer (S3500, Microtrac, York, PA, USA) with isopropanol as the medium. Scanning electron microscopy (SEM) and energy dispersive spectroscopy (EDS) analyses were performed using a field-emission scanning electron microscope (FE-SEM, JSM-7600F, JEOL, Tokyo, Japan) equipped with an energy dispersive X-ray spectrometer (Inca Oxford 350 EDS SSD, Oxford Instruments, Abingdon, UK). For the SEM powder analyses, Al_2_O_3_ and Al powders were deposited on carbon tape. For the cross-sectional analysis of the multilayers, the samples were cut, mounted in epoxy resin, ground and fine-polished with a colloidal silica suspension. Prior to the SEM analyses, all the samples were coated with a 3-nanometre-thick carbon layer using a Precision Etching and Coating System (PECS 682, Gatan, Pleasanton, CA, USA).

The X-ray diffraction (XRD) analysis was performed using a high-resolution diffractometer (X’Pert PRO, PANalytical, Almelo, The Netherlands) with Cu–Kα_1_ radiation. Diffraction patterns were recorded in the BraggBrentano geometry with a 100-channel X’Celerator detector in a 2*θ* range 10–120° with a step of 0.017° and an integration time of 100 s per step. The software X’Pert HighScore Plus 2.1, PANalytical was used to analyse the XRD patterns and to estimate the penetration depth of X-rays in the multilayer samples during XRD analysis. In the case of the multilayer sample, the X-rays penetrate all deposited layers (top Al_2_O_3_ layer, middle Al layer and bottom Al_2_O_3_ layer) and reach the Gd substrate, since the penetration depth is higher than the thickness of deposited layers. The total thickness of deposited layers is ~8 µm; while the calculated penetration depth for Al and Al_2_O_3_ at 2*θ* of 10° is ~30 µm and the value even increases with increasing 2*θ* angle.

The Topas R package (version 2.1, Bruker AXS GmbH, Karlsruhe, Germany) was employed for the Rietveld refinement and the Fundamental Parameters Approach (FPA) was used for line-profile fitting of all samples [30]. The FPA uses the geometrical properties of the diffraction experiment to build up the instrumental linewidth from first principles. It, thus, allows an explicit determination of the sample-dependent, line-broadening contributions to the peak profile, which are dominated by the microstrain and the broadening of the crystallite size [31].

The topography of the layers was analysed using atomic force microscopy (AFM) and contact stylus profilometry. The 20-micrometre line scans were acquired using an atomic force microscope (Jupiter XR, Asylum Research, Santa Barbara, CA, USA) in AC air topography mode. A Si tip on a Si/Al cantilever with a diameter of ~7 nm (AC240TS-R3, Asylum Research, Santa Barbara, CA, USA) was used for scanning. A contact stylus profilometer (DektakXT, Bruker, Karlsruhe, Germany) was used to measure 2-millimetre line scans. Then, the root-mean-square surface roughness (*R_q_*) was determined from the roughness profile obtained after high-pass filtering of the primary profile with a cut-off wavelength of 0.08 mm. 

Silver electrodes with a diameter of 0.75 mm were deposited on the top Al_2_O_3_ layer before electrical characterisation. Current density–electric field (*J–E*) measurements were performed using a Keithley 237 high-voltage-source measurement unit (Keithley Instruments, Cleveland, OH, USA). A step-like electric field in the range ±75 kV∙cm^−1^ was applied between Al layer and silver electrodes. The electrical resistivity was determined from the slope of the *J–E* curve, assuming Ohm’s law.

## 3. Results

**Figure 1:**
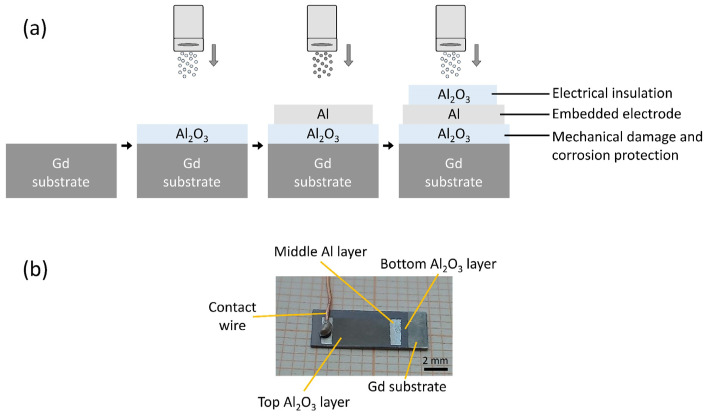
(**a**) Schematic representation of the deposition process in the multilayer fabrication. (**b**) A photograph of the multi-layered structure with an electrical contact.

We prepared an Al_2_O_3_/Al/Al_2_O_3_ multilayer structure on the surface of a magnetocaloric gadolinium element. A schematic representation and a photograph of the multilayer structure are shown in Figure 1a,b, respectively. First, the Gd substrate was almost completely covered with an Al_2_O_3_ layer, followed by the deposition of an Al layer. Then, the Al_2_O_3_ layer was deposited on top of the Al layer, keeping certain areas free to allow for electrical connections (i.e., placement of the contact wires). The first Al_2_O_3_ layer protects the Gd substrate from the environment and prevents corrosion and mechanical damage. The second Al layer is an electrically conductive electrode layer covered by the third Al_2_O_3_ layer, which electrically insulates the Al surface and completes the multilayer structure with an embedded electrode.

**Figure 2:**
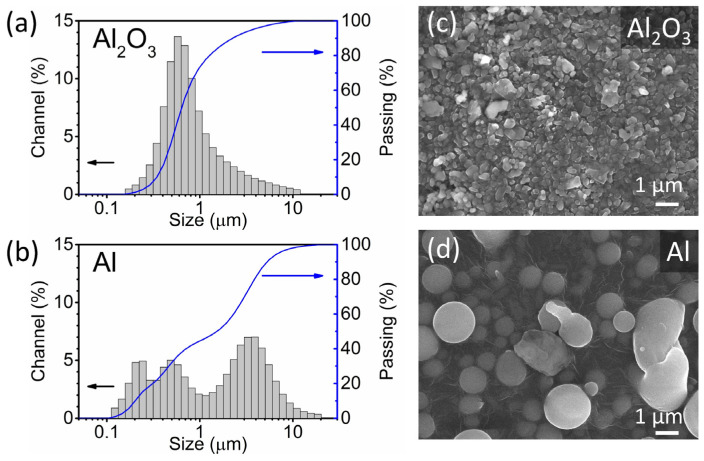
(**a**,**b**) Particle size distributions (grey) and cumulative curves (blue) determined using the laser granulometry and (**c**,**d**) scanning electron microscopy (SEM) images of Al_2_O_3_ and Al powders.

The Al_2_O_3_ and Al powders used in the deposition were analysed using laser granulometry and SEM. Laser granulometry shows a monomodal (Figure 2a) and a multi-modal (Figure 2b) particle size distribution of the Al_2_O_3_ and Al powders, respectively. The particle size range of the two powders is between 0.1 and 20 µm, and the median particle size (*d*_50_) of the Al_2_O_3_ and Al powders is 0.6 and 1.5 µm, respectively. The most abundant particle size fraction of the Al_2_O_3_ powder is represented by the peak at 0.6 µm, while the Al powder contains three peaks at 0.2, 0.5 and 3 µm. Both powders have an acceptable particle size range for the AD. In the literature, particles with sizes between a few hundred nm and a few µm are generally considered suitable for deposition and produce dense thick films with good adhesion [4,6]. According to the SEM analysis, the Al powder exhibits round particles, often with a perfect circular shape (Figure 2d). In contrast, the Al_2_O_3_ particles are irregularly shaped with smooth edges (Figure 2c).

**Figure 3:**
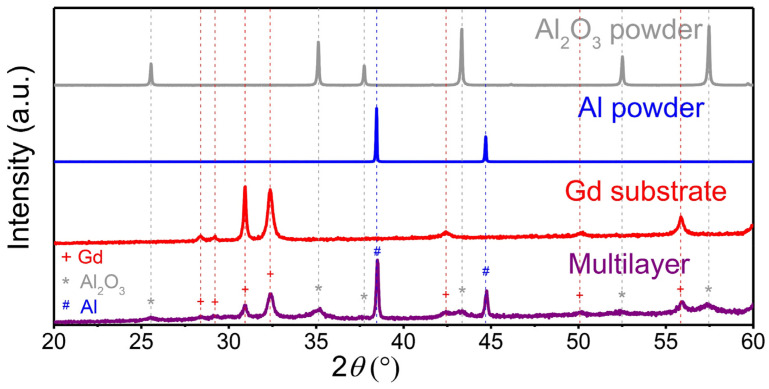
X-ray diffraction (XRD) patterns of the Al_2_O_3_ powder (grey), Al powder (blue), Gd substrate (red) and prepared multilayer sample (purple).

XRD analysis (Figure 3) was performed on the Al_2_O_3_ and Al powders, on the Gd substrate and on the multilayer sample after deposition. The XRD patterns of the Al_2_O_3_ and Al powders contained only Al_2_O_3_ (JCPDS 46-1212) and Al reflections (JCPDS 89-2769), respectively. Therefore, no significant powder contamination in the ball milling process of the Al_2_O_3_ powder was detected. The very sharp peaks indicate large crystallites (>100 nm) and no microstrain in both powders. As expected, the XRD pattern of the Gd substrate also exhibits sharp Gd reflections (JCPDS 89-2924) with no impurities. After deposition, the XRD pattern of the multilayer (purple) shows the reflections of the Al layer, the Al_2_O_3_ layer and the Gd substrate. None of the three phases underwent a phase transformation. In the multilayer, the Gd reflections (marked with a red cross) did not undergo any peak shift or change in the peak shape. Only the intensity of the Gd decreased, since the substrate was covered by Al_2_O_3_ and Al layers. After the deposition, the multilayer sample exhibits peak broadening of the Al_2_O_3_ (marked with a grey asterisk) and Al (marked with blue dash) reflections, indicating a decrease in the crystallite size and/or an increase in the microstrain due to the fragmentation of the colliding powder particles in the AD process [4,32]. To quantitatively evaluate the crystallite size and microstrain in the Al_2_O_3_ and Al deposited layers, we performed a Rietveld refinement. The calculated crystallite size and microstrain in the Al_2_O_3_ layers are 22 ± 4 nm and 0.46 ± 0.14%, respectively. On the other hand, the crystallites in the Al layers are larger, i.e., 98 ± 8 nm, while the microstrain is almost insignificant, i.e., 0.04 ± 0.01%. In conclusion, the XRD results show that the deposition of Al_2_O_3_ leads to a much larger decrease in crystallite size and an increase in microstrain compared to Al.

Previously, it was reported that a significant reduction in the crystallite size and an increase in the microstrain after the deposition of an oxide powder are necessary conditions for successful film deposition [33]. According to our XRD analysis, the same hypothesis can be valid for the AD of ceramic Al_2_O_3_, but not completely for the AD of metallic Al. Both Al_2_O_3_ and Al powders formed consolidated layers, but only the ceramic Al_2_O_3_ powder obtained significant peak broadening, indicating that the cracking and fragmentation of particles predominate in the deposition mechanism. On the other hand, the deposition mechanism of ductile metals is different from that of ceramics. The less intense XRD peak broadening of Al indicates that plastic deformation is more prevalent in the deposition mechanism of metals.

**Figure 4:**
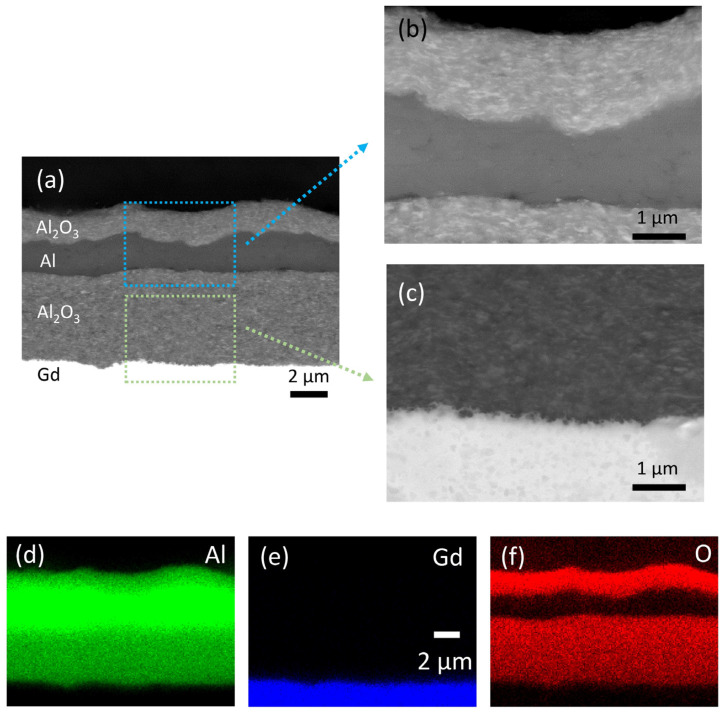
(**a**) Scanning electron microscopy (SEM) analysis of the multilayer structure in cross-section. Panels (**b**,**c**) show the magnified areas marked in (**a**). Energy dispersive spectroscopy (EDS) elemental mapping images are showing the distribution of (**d**) Al, (**e**) Gd and (**f**) O.

The SEM and EDS analyses of the multilayer structure in cross-section are shown in Figure 4. All the deposited layers are very dense without any visible pores (Figure 4a). The thickness of the deposited Al_2_O_3_, Al and Al_2_O_3_ layers (bottom-up) is 5.0, 1.7 and 1.6 µm, respectively. Apparently, the roughness of the interfaces increases with the number of increasing layers. However, a sufficient layer thickness ensures good connectivity of the deposited layers. The interface between the layers is well defined (enlarged SEM images in Figure 4b,c), indicating good adhesion without any reactions. An additional adhesion test revealed no peeling off or delamination of the deposited layers (Supplementary material: Figure S1). The EDS map scans (Figure 4d–f) of the multilayer show a typical elemental distribution of the layers, which confirms no reaction between the layers.

**Figure 5:**
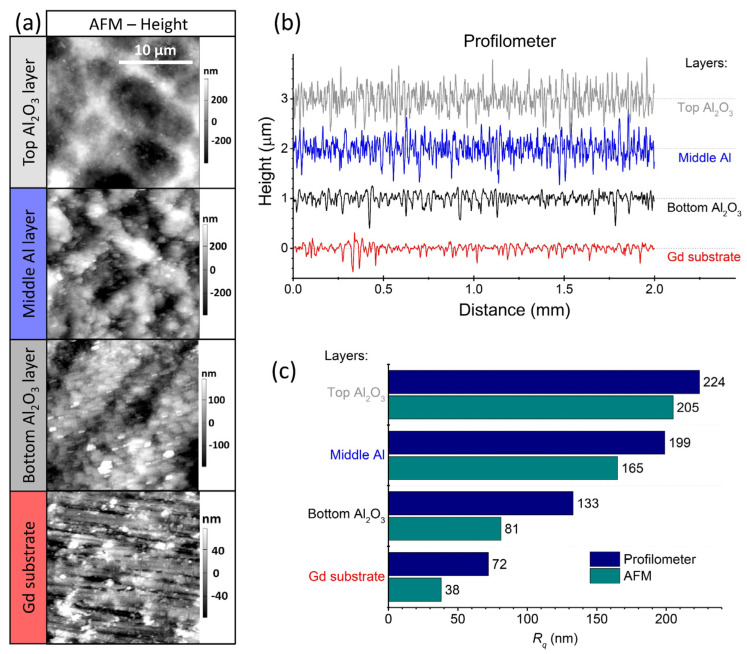
(**a**) Atomic force microscopy (AFM) map scans and (**b**) contact-profilometer line scans of the deposited layers, including the Gd substrate. The corresponding *R_q_* values of the two methods, AFM (cyan) and contact profilometry (dark blue), are shown in (**c**).

The surface roughness of the deposited layers including the Gd substrate was evaluated using AFM and contact profilometry. The AFM map scans and the contact-profilometry line scans are shown in Figure 5a,b, respectively. A comparison of the root-mean-square surface roughness (*R_q_*) between the two measurement methods is shown in Figure 5c. As expected, the values obtained with AFM are lower than those obtained with contact profilometry due to the hundred-times-smaller scanned area (20-micrometre lines in the case of the AFM and 2-millimetre lines in the case of the contact profilometry). However, the same trend is observed for both methods. The *R_q_* is lowest for the Gd substrate and increases with each deposited layer. The *R_q_* of the top Al_2_O_3_ layer is about 200 nm.

**Figure 6:**
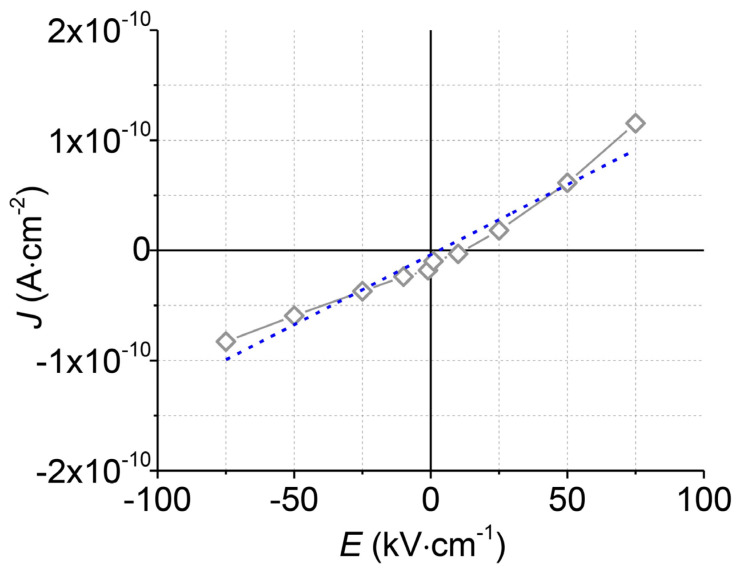
The current density (*J*) vs. electric field (*E*) curve (grey) of the top Al_2_O_3_ layer and its linear approximation (blue).

To test the electrical insulation of the upper Al_2_O_3_ layer, measurements of the current density (*J*) versus the electric field (*E*) were performed (Figure 6). The upper Al_2_O_3_ layer withstands high electric fields (75 kV·cm^−1^) without an electrical breakdown. The calculated electrical resistivity at room temperature is very high (7.7 × 10^12^ Ω·m) and corresponds to the resistivity of the commercially available Al_2_O_3_ ceramics for electrical insulation [34,35].

## 4. Conclusions

We used the AD method to prepare an Al_2_O_3_/Al/Al_2_O_3_ multilayer composite on a Gd substrate. The complete room-temperature processing of the AD enabled the integration of metallic and ceramic materials that are otherwise incompatible at high temperatures. Inexpensive commercial powders with appropriate micrometre-sized particles were used for the successful film deposition. The SEM analysis revealed a dense multilayer with a defect-free microstructure and good intra-layer connectivity. In addition, the top Al_2_O_3_ dielectric layer provides excellent electrical resistance, which is required for reliable electric field application. In summary, we have shown that the fabrication of ceramic–metal multilayers using AD is feasible and represents a simple, reliable and cost-effective approach to functionalise and protect existing substrates. For example, the deposited Al_2_O_3_/Al/Al_2_O_3_ multilayers on the Gd substrate provide an excellent starting point for the development of future electrowetting-on-dielectric devices.

## Figures and Tables

**Table 1:** Process parameters used during the AD.

Process Parameters	Al_2_O_3_ Powder	Al Powder
Carrier gas species	N_2_	
Nozzle geometry (slit size)	(0.5 × 10) mm^2^	
Distance between nozzle and substrate	5 mm	
Sweep speed	5 mm·s^−1^	
Gas flow rate	4 L·min^−1^	2 L·min^−1^
Pressure in aerosol chamber	180 mbar	120 mbar
Pressure in deposition chamber	2 mbar	1 mbar

## Data Availability

The data presented in this study are available upon request from the corresponding author.

## Supplementary Materials

The following are available online at https://www.mdpi.com/article/10.3390/ma14164548/s1, Figure S1: Images of the sample surface (top Al_2_O_3_ layer) taken with an optical light microscope. (a) before and (b) after the adhesion test.
