# Supplementary material for: Investigating the Feasibility of Preparing Metal–Ceramic Multi-Layered Composites Using Only the Aerosol-Deposition Technique
Source: Materials (Basel). 2021 Aug 13;14(16):4548. doi: 10.3390/ma14164548 (PMC8400038; doi:10.3390/ma14164548)
Supplement: Supplementary file 1 [file materials-14-04548-s001.zip › materials-1307515-supplementary.pdf]

materials MDPI

Supplementary Material

Investigating the Feasibility of Preparing Metal–Ceramic Multi-Layered Composites Using Only the Aerosol-Deposition Technique

Matej Sadl [1,2], Urban Tomc [3] and Hana Ursic [1,2,*]

1 Electronic Ceramics Department, Jožef Stefan Institute, Jamova cesta 39, 1000 Ljubljana, Slovenia; matej.sadl@ijs.si
2 Jožef Stefan International Postgraduate School, Jamova cesta 39, 1000 Ljubljana, Slovenia
3 Laboratory for Refrigeration and District Energy, Faculty of Mechanical Engineering, University of Ljubljana, Aškerčeva cesta 6, 1000 Ljubljana, Slovenia; urban.tomc@fs.uni-lj.si
* Correspondence: hana.ursic@ijs.si

S1: Adhesion test

To evaluate the adhesion between the deposited layers, an adhesive tape test was performed on the multilayer sample. The surface of the top Al_2O_3 layer was examined with an optical light microscope (Type 307 – 107.003, Leitz Wetzlar, Germany) before and after the test. For the adhesion test, a adhesive tape (Scotch Tape, 3M) was pressed firmly against the examined sample surface and then quickly peeled off at an angle perpendicular to the sample surface. The sample surface was optically examined immediately after the adhesive tape was removed (Figure S1).

After removal of the adhesive tape, the analysis revealed no delamination of deposited layers. Minor differences in the surface before and after the adhesion test can be attributed to the removal of surface contaminants.

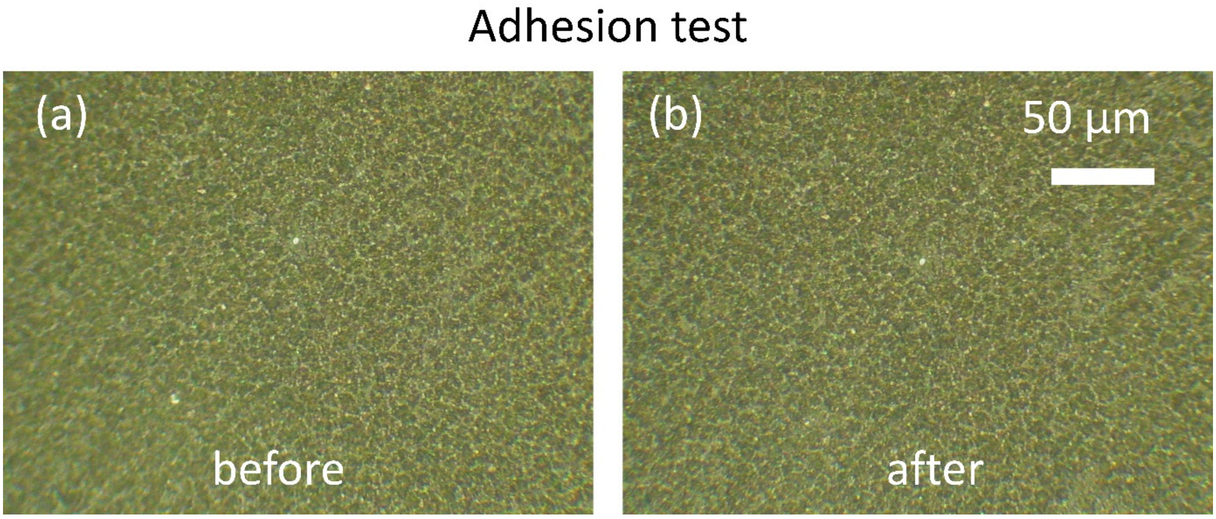

Figure S1. Images of the sample surface (top Al_2O_3 layer) taken with an optical light microscope. (a) before and (b) after the adhesion test.
